# Fluorite–Perovskite Heterointerface Engineering Boosts CO_2_ Electrolysis Performance in Solid Oxide Electrolysis Cells

**DOI:** 10.3390/ma19194158

**Published:** 2026-09-29

**Authors:** Guanyu Chen, Bo Yin, Di Zhang, Yifeng Zheng, Sheng Cui

**Affiliations:** 1 College of Materials Science and Engineering, Nanjing Tech University, No. 30 Puzhu Road(S), Nanjing 211816, China; chenguanyu@njtech.edu.cn (G.C.); 15255741742@163.com (D.Z.); scui@njtech.edu.cn (S.C.); 2 Morgan Thermal Ceramics (Shanghai) Co., Ltd., No. 18 Kang An Road, Kang Qiao Industrial Zone Pudong, Shanghai 201315, China; bob.yin@morganplc.com; 3 Morgan Advanced Materials plc (Group) Yixing Morgan Thermal Ceramics Co., Ltd., No. 2 Beidan Road, Ceramic Industrial Park, Dingshu Town, Yixing 214221, China

**Keywords:** solid oxide electrolysis cells, CO_2_ electrolysis, fuel electrode, infiltration, fluorite–perovskite heterointerface, Pr-doped ceria

## Abstract

Solid oxide electrolysis cells (SOECs) provide a promising route for renewable-electricity storage and CO_2_ conversion into value-added chemicals and fuels, yet their high-temperature operating environment requires the development of efficient and stable fuel electrode materials. Sr_0.9_Ti_0.45_Fe_0.5_W_0.05_O_3−δ_ (STFW) perovskite material fabricated in our previous work shows a promising SOEC fuel electrode and is expected to be further optimized. Herein, we develop a heterointerface engineering strategy by infiltrating fluorite-type Pr_0.1_Ce_0.9_O_2−δ_ (PCO) into the porous STFW fuel electrode, constructing a highly active PCO-STFW composite fuel electrode. The optimized 30PCO-STFW (30 μL PCO) electrode achieves a polarization resistance of 0.14 Ω cm^2^ at 800 °C. A single cell employing 30PCO-STFW as the fuel electrode delivers an electrolysis current density of 1.33 A cm^−2^ at 800 °C and 1.5 V, which is 23% higher than that of the STFW cell. Moreover, the 30PCO-STFW cell maintains relatively stable operation at 800 °C and 1.2 V for 70 h. This study demonstrates that PCO-enabled fluorite–perovskite interfacial engineering effectively regulates the surface reaction environment of STFW fuel electrodes, providing an effective strategy for improving CO_2_ electrolysis performance in SOECs.

## 1. Introduction

The continuous increase in CO_2_ emissions has stimulated intensive efforts toward carbon capture, conversion, and utilization for sustainable energy systems [1,2,3]. Among various CO_2_ conversion technologies, solid oxide electrolysis cells (SOECs) offer a particularly attractive route because high-temperature operation enables the efficient coupling of renewable electricity and thermal energy for CO_2_-to-CO conversion [4,5]. During CO_2_ electrolysis, CO_2_ is adsorbed and reduced to CO at the fuel electrode of SOEC, while the generated O^2−^ species are transported through the dense electrolyte to the air electrode, where O_2_ is released. Therefore, the fuel electrode plays a central role in determining CO_2_ adsorption/activation, charge transfer, oxygen-species transport, and gas diffusion; its reaction kinetics strongly influence the overall polarization loss and electrolysis performance of SOECs.

Ni-based cermet fuel electrodes have been widely employed because of their high electronic conductivity and catalytic activity, but their application in direct CO_2_ electrolysis is limited by redox instability, particle agglomeration, and carbon deposition [6,7,8,9,10]. Perovskite oxides provide an alternative platform due to their structural tunability, redox stability, coking resistance, and mixed ionic–electronic transport characteristics. In particular, SrTiO_3_-based perovskites exhibit excellent structural stability under CO_2_-containing atmospheres, although their relatively sluggish surface reaction kinetics remain a major limitation [11,12]. Defect engineering through A-site deficiency and B-site substitution has been widely employed to regulate lattice defects, transition-metal valence states, and oxygen nonstoichiometry [13,14]. Our previous work demonstrated that the combination of A-site Sr deficiency and B-site W doping effectively improves the physicochemical and electrochemical properties of SrTi_0.5_Fe_0.5_O_3−δ_, with the developed Sr_0.9_Ti_0.45_Fe_0.5_W_0.05_O_3−δ_ (STFW) exhibiting reasonable CO_2_ electrolysis performance [15]. Nevertheless, bulk defect regulation alone cannot fully address the sluggish surface and interfacial reactions, because CO_2_ conversion predominantly occurs within the electrochemically active surface and electrode/electrolyte interfacial regions.

Surface infiltration strategy provides an effective means of overcoming this limitation by introducing nanoscale functional phases into a preformed porous electrode without substantially altering the host backbone. The resulting nanoscale interfacial contact can increase the density of surface reaction sites and facilitate charge transfer and oxygen-species exchange [16,17,18,19]. However, the effectiveness of infiltration strongly depends on the physicochemical properties and loading of the infiltrated phase. Insufficient loading may provide limited interfacial modification, whereas excessive deposition can partially block gas-transport pathways or disrupt electronic transport continuity; high-temperature particle coarsening may further reduce the beneficial nanoscale interfacial area [20,21,22,23,24]. Therefore, the appropriate surface modifier should combine high surface reactivity, favorable oxygen-exchange capability, and structural compatibility with the perovskite backbone.

Ceria-based fluorite oxides are attractive candidates for such surface modification because of their reversible Ce^4+^/Ce^3+^ redox chemistry, oxygen storage/release capability, and favorable surface reactivity [25,26]. Previous studies have demonstrated that nanoscale CeO_2_-based phases can promote CO_2_-related surface reactions and improve electrochemical performance when incorporated into oxide fuel electrodes. Compared with undoped ceria, Pr-doped ceria (Pr_0.1_Ce_0.9_O_2−δ_, PCO) exhibits more complex surface defect chemistry arising from the multivalent nature of Pr [27]. Experimental and theoretical studies have shown that Pr incorporation can facilitate oxygen-vacancy formation and migration, while operando spectroscopy revealed a pronounced enrichment of Pr^3+^ in the near-surface region of PCO [28,29,30,31,32]. These characteristics make PCO a promising surface functional phase for regulating oxygen exchange and the local reaction environment during high-temperature CO_2_ electrolysis.

In the present work, we construct a PCO/STFW fluorite–perovskite composite fuel electrode by infiltrating fluorite-type PCO into the porous STFW backbone. In this architecture, STFW serves as the structurally stable perovskite backbone and provides the primary electronic transport network, whereas the fluorite-type PCO functions as a surface modifier to regulate the near-surface oxygen environment and create extensive fluorite–perovskite interfacial contact. The PCO loading is further optimized to balance the beneficial surface modification against the preservation of gas-transport pathways and electronic transport continuity. This design integrates bulk defect regulation of STFW with surface heterointerface engineering, aiming to overcome the surface and interfacial kinetic limitations that remain after conventional bulk compositional modification. Unlike conventional ceria-based infiltration strategies that primarily introduce an additional ionic-conducting or catalytic phase, the present PCO modification is designed to selectively regulate the near-surface oxygen environment and surface-exchange kinetics of STFW through the multivalent Pr-containing fluorite phase. The effect of PCO infiltration on the phase structure, microstructure, electrical transport, thermomechanical compatibility, surface oxygen chemistry, oxygen-exchange kinetics, and CO_2_ electrolysis behavior of STFW was systematically investigated.

## 2. Experimental Procedure

### 2.1. Powder Synthesis and Cell Fabrication

STFW powder was synthesized by a conventional solid-state reaction method. Stoichiometric amounts of SrCO_3_, TiO_2_, Fe_2_O_3_, and WO_3_ were used as starting materials and ball-milled in anhydrous ethanol with zirconia milling media at 250 rpm for 24 h to ensure homogeneous mixing. After drying, grinding, and sieving, the mixed powders were calcined at 1150 °C for 10 h in air. The details can be referred to in our previous study [15].

The PCO precursor solution was prepared by a nitrate complexation method. Stoichiometric amounts of Pr(NO_3_)_3_·6H_2_O and Ce(NO_3_)_3_·6H_2_O were dissolved in 100 mL of deionized water. Citric acid was then added as a complexing agent, with a molar ratio of citric acid to total metal ions of 1.5:1. The solution was magnetically stirred until a homogeneous and transparent PCO precursor solution was obtained, with a total metal-ion concentration of 0.1 mol L^−1^.

LSGM (La_0.9_Sr_0.1_Ga_0.8_Mg_0.2_O_3_) electrolyte pellets were fabricated by dry pressing and high-temperature sintering. LSGM powder was uniaxially pressed at 200 MPa and sintered at 1450 °C for 5 h in air. The sintered electrolyte pellets were polished with sandpapers to obtain flat surfaces and controlled electrolyte thickness. The STFW fuel electrode slurry was prepared by mixing STFW powder with an organic vehicle composed of ethyl cellulose and terpineol. After sufficient grinding or ball milling, a homogeneous slurry suitable for screen printing was obtained. The STFW slurry was then screen printed onto one side of the LSGM electrolyte and calcined at 950 °C for 2 h to form a porous STFW fuel electrode backbone.

PCO was introduced into the porous STFW fuel electrode backbone by a solution infiltration method. Typically, 5 μL of the PCO precursor solution was dropped onto the pre-sintered STFW fuel electrode using a micropipette, allowing the precursor solution to fully penetrate into the porous electrode framework. The infiltrated electrode was then calcined at 950 °C for 2 h. The PCO loading amount was controlled by adjusting the number of infiltration cycles, giving total infiltration volumes of 20, 30, and 40 μL. The corresponding composite fuel electrodes were denoted as 20PCO-STFW, 30PCO-STFW, and 40PCO-STFW, respectively. Based on the precursor concentration of 0.1 mol L^−1^, the nominal PCO amounts corresponding to total infiltration volumes of 20, 30, and 40 μL were approximately 0.344, 0.517, and 0.689 mg, respectively. Considering an effective electrode area of 0.5 cm^2^, the corresponding nominal areal PCO loadings were approximately 0.689, 1.033, and 1.378 mg cm^−2^, respectively.

For the fabrication of symmetrical cells, the fuel electrode slurry was screen-printed onto both sides of the dense LSGM electrolyte pellet to form identical electrode layers. The printed cells were subsequently calcined at 950 °C for 2 h in air to obtain porous fuel electrodes with good adhesion to the electrolyte. The effective electrode area on each side was controlled at 0.5 cm^2^. For STFW symmetrical cells, STFW slurry was directly applied to both sides of the LSGM electrolyte. For PCO-modified symmetrical cells, the PCO precursor solution was infiltrated into the pre-sintered STFW electrodes following the same infiltration procedure described above to obtain 20PCO-STFW, 30PCO-STFW, and 40PCO-STFW electrodes. Before electrochemical measurements, silver paste was applied to both electrode surfaces as current collectors and calcined at 750 °C for 10 min.

Single cells were fabricated using LSGM as the electrolyte, STFW or PCO-STFW as the fuel electrode, and LSCF-GDC as the air electrode. The LSCF-GDC air-electrode slurry was prepared by mixing LSCF and GDC powders with an organic vehicle and then screen-printed onto one side of the LSGM electrolyte, while the fuel electrode slurry was printed on the opposite side. After calcination at the corresponding temperatures, porous electrode layers were formed. Before electrochemical testing, silver paste was applied onto the electrode surfaces as current collectors and calcined at 750 °C for 10 min to ensure good current collection and electrical contact.

### 2.2. Characterization and Measurements

X-ray diffraction (XRD) was used to analyze the phase structures and chemical compatibility of STFW, PCO-STFW composite powders, and electrode/electrolyte mixtures. XRD measurements were performed using Cu Kα radiation over a 2θ range of 20–80°. Rietveld refinement was conducted on the XRD data to further determine the phase composition of the PCO-STFW composite powder. To evaluate the high-temperature chemical compatibility between the PCO-STFW fuel electrode and the LSGM electrolyte, PCO-STFW and LSGM powders were mixed at a mass ratio of 1:1, calcined at 1000 °C for 2 h in air, and then characterized by XRD. Scanning electron microscopy (SEM) was employed to observe the electrode surface morphology, cross-sectional microstructure, and fuel electrode/electrolyte interface, thereby clarifying the effect of PCO infiltration on the porous STFW backbone and interfacial adhesion. After the durability test, SEM was also used to examine the electrode cross-section and surface morphology to evaluate structural evolution during the 70 h CO_2_ electrolysis test.

The electrical conductivities of STFW and PCO-STFW samples were measured by a four-probe direct-current method in air over the temperature range of 300–800 °C. Thermal expansion behavior was measured using a dilatometer in air from 40 to 800 °C, and the average thermal expansion coefficient was calculated from the dimensional change in the sample to assess thermal compatibility between the fuel electrode and the LSGM electrolyte.

Electrochemical impedance spectroscopy (EIS) was used to evaluate the polarization resistance of STFW and PCO-STFW fuel electrodes with different PCO loading amounts. Symmetrical-cell measurements were carried out under a 70% CO_2_/30% CO atmosphere at 650–800 °C with an AC perturbation amplitude of 10 mV. To compare the reaction kinetics of different electrodes, the ohmic resistance was subtracted from the impedance spectra, and the arc diameter was used to determine the total polarization resistance. The total polarization resistance obtained from the symmetrical cell was divided by two to obtain the polarization resistance of a single electrode and normalized to the electrode area. Arrhenius fitting was performed based on the Rp values obtained at different temperatures to determine the apparent activation energy. In addition, distribution of relaxation times (DRT) analysis was performed on the EIS data using a MATLAB (R2024b)-based fitting tool to resolve electrochemical responses occurring over different characteristic time scales. The resulting DRT profiles were divided into high-frequency (HF), intermediate-frequency (IF), and low-frequency (LF) regions according to their characteristic frequency ranges. Because DRT analysis alone cannot uniquely identify individual elementary reaction steps, the assignments of these frequency regions were regarded as literature-guided interpretations based on their characteristic frequencies and previous SOC studies.

Electrical conductivity relaxation (ECR) measurements were performed at 650 °C using a four-probe DC method with Ag wires. Rectangular bar-shaped samples with dimensions of 4.2 cm × 0.506 cm × 0.156 cm were used. During the measurements, the total gas flow rate was maintained at 100 mL min^−1^ for all atmospheres. The samples were initially equilibrated in N_2_ until a stable conductivity was reached, after which the atmosphere was switched to air and subsequently to humidified air containing 3 vol.% H_2_O. The humidified air was obtained by passing air through a water bubbler. The conductivity response was continuously recorded during each atmosphere-switching process until a new equilibrium state was established. The normalized conductivity relaxation was calculated as $g\left(t\right)=\frac{\sigma_{t}-\sigma_{0}}{\sigma_{\infty }-\sigma_{0}}$, where *σ*_0_, *σ_t_*, and *σ_∞_* represent the initial, transient, and final equilibrium conductivities, respectively. The conductivity relaxation response from the initial N_2_ atmosphere to the final humidified-air atmosphere was recorded and fitted using MATLAB (R2024b). The chemical diffusion coefficient (*D_chem_*) and chemical surface-exchange coefficient (*K_chem_*) were extracted by fitting the normalized relaxation curves using the analytical solution of Fick’s second law for a rectangular specimen under finite surface-exchange boundary conditions.

Single-cell CO_2_ electrolysis performance was evaluated using current–voltage (I–V) curves at 700–800 °C. STFW or PCO-STFW was used as the fuel electrode, LSCF-GDC as the air electrode, and LSGM as the electrolyte. The fuel electrode side was supplied with a CO_2_-containing reaction gas, while the air-electrode side was exposed to air. The effect of PCO infiltration on practical CO_2_ electrolysis performance was assessed by comparing the I-V curves of STFW and PCO-STFW single cells at different temperatures. Stepwise potentiostatic testing was subsequently performed at 800 °C to examine the current response of the single cell under different applied voltages. Short-term operational stability testing was conducted at 800 °C and 1.2 V by continuously recording the current density for 70 h. After testing, Raman spectroscopy was performed on the fuel electrode to identify possible SrCO_3_, D-band, or G-band signals associated with carbonate formation or carbon deposition.

## 3. Results and Discussion

### 3.1. Material Characterization

**Figure 1:**
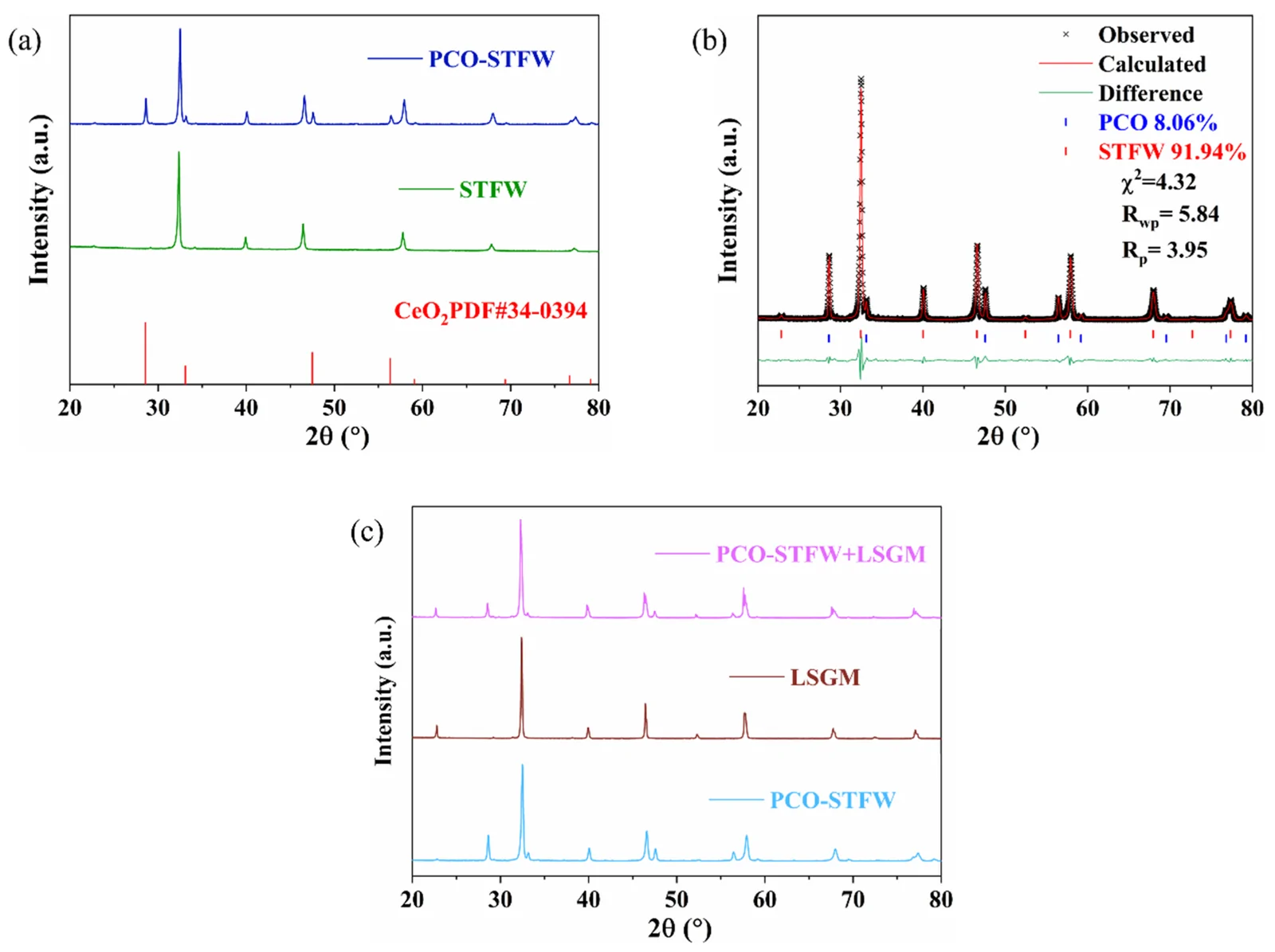
(**a**) XRD patterns of STFW and PCO-STFW, together with the standard diffraction pattern of fluorite CeO_2_. (**b**) Rietveld refinement of the 30PCO-STFW composite. (**c**) XRD patterns of PCO-STFW, LSGM, and their mixture after heat treatment at 1000 °C for 2 h.

To evaluate the effect of PCO infiltration on the phase structure and interfacial stability of the STFW fuel electrode, XRD measurements were first performed on STFW and PCO-STFW composite powders. As shown in Figure 1a, the pristine STFW sample exhibits characteristic diffraction peaks of a perovskite oxide, confirming the successful formation of the STFW main phase. After PCO infiltration and subsequent heat treatment, the major diffraction peaks of the PCO-STFW sample remain consistent with those of the STFW perovskite phase, indicating that the infiltration process does not disrupt the crystal structure of STFW. Meanwhile, additional diffraction signals matching the standard fluorite CeO_2_ phase are observed in the PCO-STFW sample. Since Pr_0.1_Ce_0.9_O_2−δ_ shares the same fluorite structure as CeO_2_, this result supports the successful introduction of the fluorite-type PCO phase into the STFW electrode. No additional impurity peaks apart from those assigned to the STFW perovskite phase and the PCO fluorite phase are detected, suggesting that no obvious secondary reaction occurs between PCO and STFW within the detection limit of XRD under the present heat-treatment conditions and that the two phases can coexist stably.

To further quantify the phase composition of the PCO-STFW composite powder, Rietveld refinement was performed on the XRD pattern, as shown in Figure 1b. The calculated pattern shows acceptable agreement with the experimental data, with R_wp_ = 5.84%, R_p_ = 3.95%, and χ^2^ = 4.32. Quantitative analysis reveals that the STFW and PCO phases account for 91.94 wt.% and 8.06 wt.% of the composite powder, respectively, further demonstrating that PCO coexists with STFW as an independent fluorite phase after infiltration and heat treatment. The results indicate that the PCO infiltration strategy can construct a stable PCO/STFW fluorite–perovskite composite structure while preserving the structural integrity of the STFW perovskite backbone.

The chemical compatibility between the PCO-STFW composite electrode and the LSGM electrolyte was further examined by mixing PCO-STFW and LSGM powders at a mass ratio of 1:1 followed by calcination at 1000 °C for 2 h in air. As shown in Figure 1c, all diffraction peaks of the calcined mixture can be assigned to the intrinsic phases of PCO-STFW and LSGM, with no additional impurity diffraction peaks attributable to secondary reaction products. This result indicates good chemical compatibility between the PCO-STFW composite electrode and LSGM under the present heat-treatment conditions.

**Figure 2:**
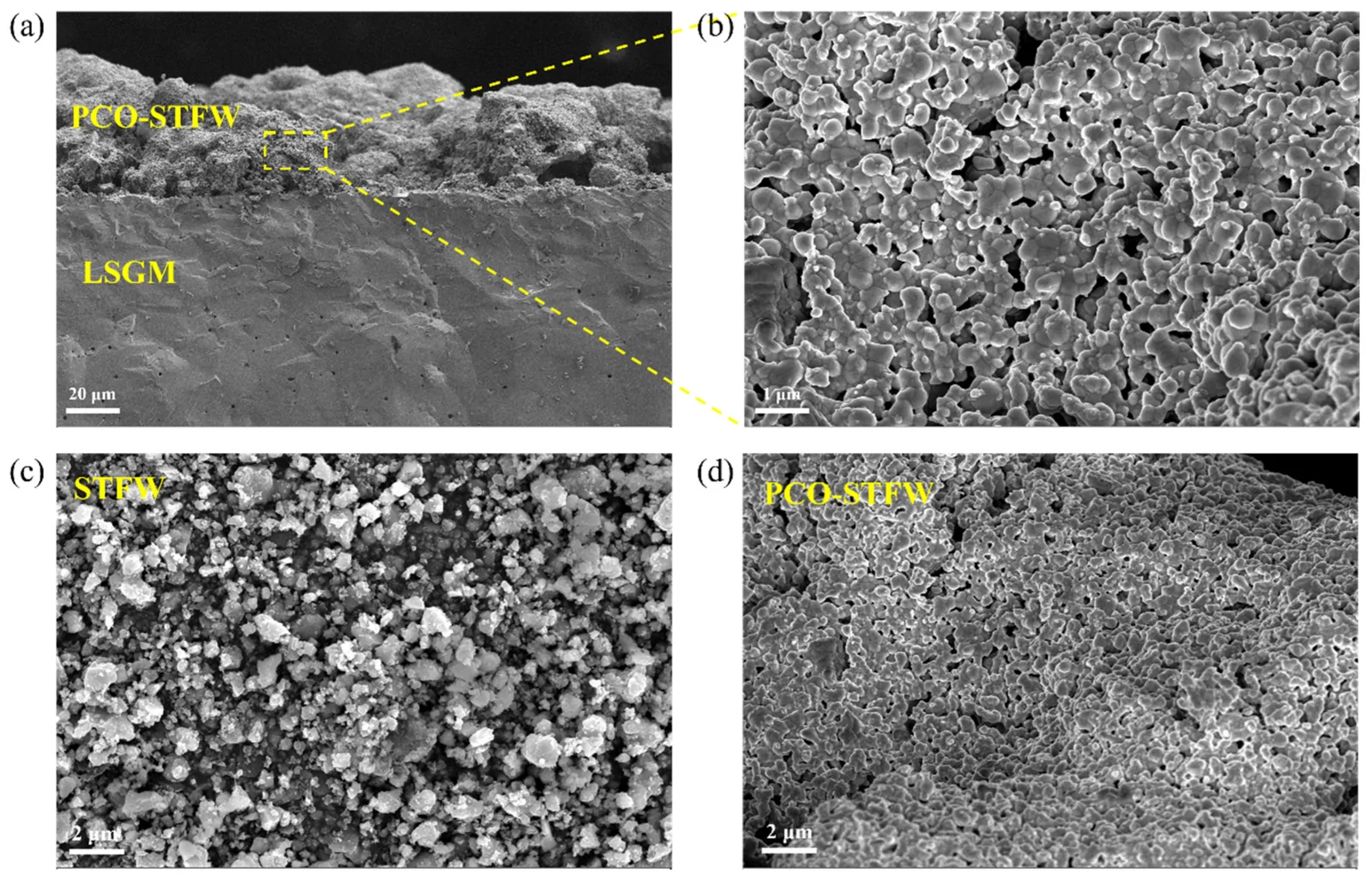
(**a**) Cross-sectional microstructure of the interface between the fuel electrode and electrolyte in the single cell; (**b**) enlarged view of the local electrode microstructure; (**c**) microstructure of the STFW fuel electrode; (**d**) microstructure of the PCO-STFW fuel electrode.

SEM is subsequently employed to examine the influence of PCO infiltration on the microstructure of the STFW fuel electrode. As shown in Figure 2a, the PCO-STFW electrode maintains contact with the LSGM electrolyte over most of the observed interface, although local interfacial irregularities and dark gaps are visible. No large-scale delamination is observed within the examined region. To further reveal the local morphological features of the PCO-STFW fuel electrode, a selected region was enlarged for detailed observation. As shown in Figure 2b, the PCO-STFW electrode exhibits an interconnected porous morphology with open pore channels, which provides pathways for gas transport within the electrode. Fine surface features are also observed after infiltration. However, SEM morphological contrast alone does not allow unambiguous distinction between the PCO and STFW phases. Therefore, these morphological features are interpreted together with the XRD results confirming the coexistence of fluorite-type PCO and perovskite STFW phases.

Figure 2c,d compare the surface morphologies of the STFW electrode before and after PCO infiltration. The STFW electrode is mainly composed of irregularly stacked particles with a porous morphology. After PCO infiltration, the overall porous electrode architecture is largely preserved, while finer surface features become apparent. Because the PCO and STFW phases cannot be distinguished unambiguously from SEM contrast alone, these surface features are not directly assigned to a specific phase. Nevertheless, when considered together with the XRD evidence for the coexistence of fluorite-type PCO and perovskite STFW phases, the morphological evolution is consistent with successful PCO incorporation into the porous STFW electrode. Such surface modification may increase interfacial contact within the composite electrode and contribute to the subsequent improvement in electrochemical reaction kinetics [16,17,18,19,20,21,22,23,24].

**Figure 3:**
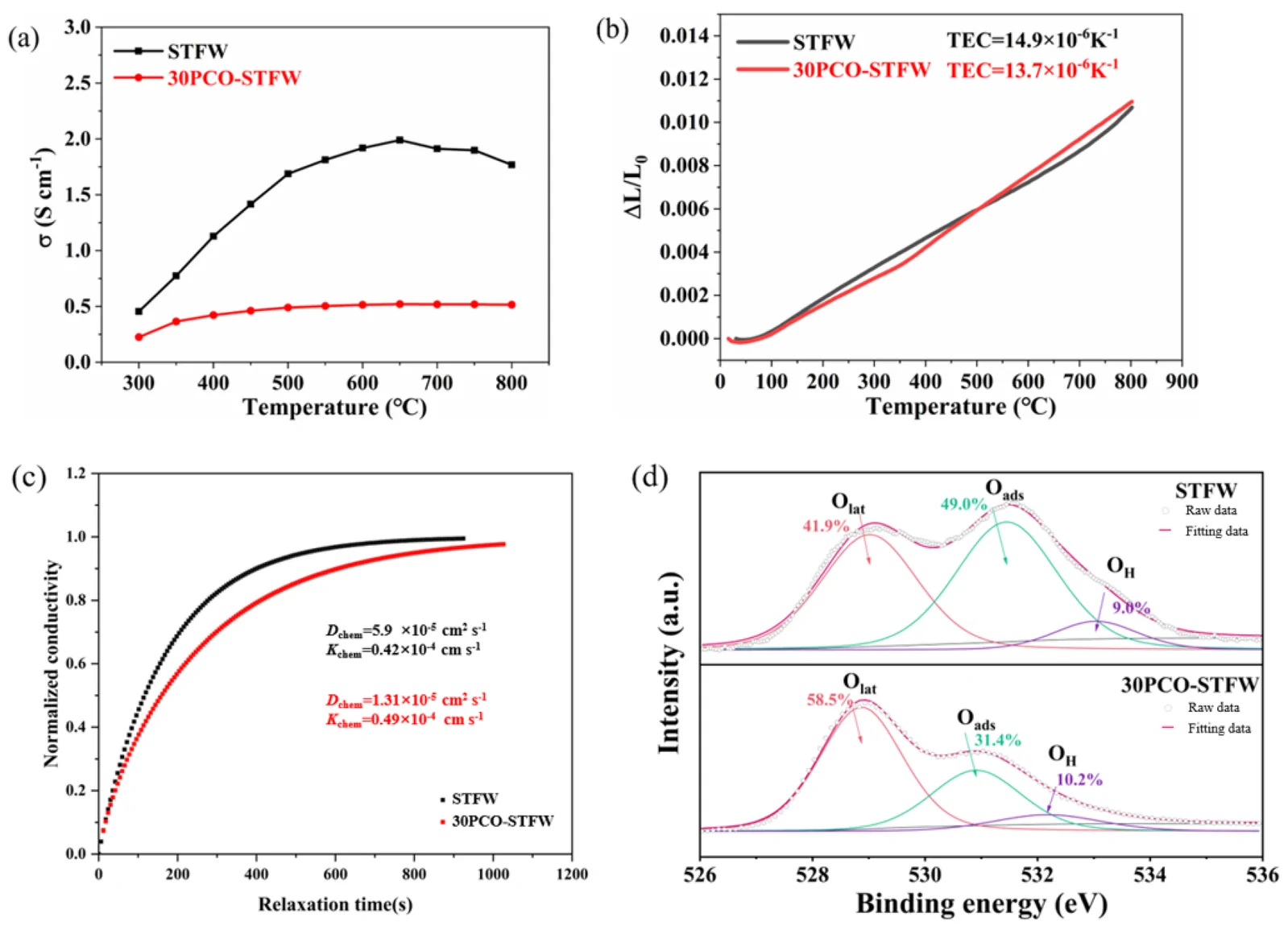
(**a**) Electrical conductivity; (**b**) thermal expansion behavior; (**c**) electrical conductivity relaxation; (**d**) O 1s XPS spectra of STFW and PCO−STFW.

Following the structural and microstructural analyses, the electrical transport properties, thermomechanical behavior, oxygen-exchange kinetics, and surface oxygen chemistry of STFW and the optimized 30PCO-STFW composite were further investigated. These physicochemical properties provide insight into whether the enhanced electrochemical performance originates from improved bulk transport or from modification of the surface and interfacial reaction environment. Figure 3a compares the electrical conductivities of STFW and 30PCO-STFW in air over the temperature range of 300–800 °C. The conductivity of pristine STFW increases from approximately 0.45 S cm^−1^ at 300 °C to a maximum of approximately 1.99 S cm^−1^ at 650 °C, followed by a moderate decrease at higher temperatures. The initial increase reflects thermally activated charge transport, whereas the decrease above 650 °C may be associated with high-temperature oxygen loss and the resulting variation in charge-carrier concentration [33,34,35]. A similar temperature dependence is observed for 30PCO-STFW, although its conductivity remains lower than that of pristine STFW throughout the investigated temperature range, increasing from approximately 0.22 S cm^−1^ at 300 °C to approximately 0.52 S cm^−1^ at 650–800 °C. The reduced conductivity can be reasonably associated with the introduction of the PCO phase and the modification of the electronically conductive STFW network. Nevertheless, an interconnected porous composite framework is retained after infiltration, which helps maintain continuous transport pathways within the electrode. More importantly, the lower conductivity of 30PCO-STFW indicates that its enhanced CO_2_ electrolysis performance does not originate from improved bulk electronic conduction but is instead primarily associated with accelerated surface and interfacial reaction kinetics.

The thermal expansion behavior of the two materials is presented in Figure 3b. Both samples exhibit continuous and smooth dimensional changes over the investigated temperature range, without any abrupt variation indicative of a pronounced structural transition. The average thermal expansion coefficient (TEC) decreases from 14.9 × 10^−6^ K^−1^ for STFW to 13.7 × 10^−6^ K^−1^ for 30PCO-STFW. The slightly reduced TEC may be beneficial for limiting thermomechanical stress during thermal cycling and indicates that PCO incorporation does not introduce severe thermomechanical incompatibility. This result is consistent with the intimate PCO-STFW/LSGM interfacial contact observed by SEM.

Electrical conductivity relaxation (ECR) measurements were further conducted to distinguish the effects of PCO infiltration on surface oxygen exchange and bulk chemical diffusion. As shown in Figure 3c, both samples gradually approach a new equilibrium conductivity state after atmosphere switching, although 30PCO-STFW requires a longer relaxation time than pristine STFW.

Quantitative fitting yields a chemical surface-exchange coefficient (K_chem_) of 0.42 × 10^−4^ cm s^−1^ for STFW and 0.49 × 10^−4^ cm s^−1^ for 30PCO-STFW. Thus, the fitted K_chem_ value shows only a modest increase after PCO modification. Because the difference between these two fitted values is relatively small and independent replicate measurements were not performed, no statistical significance is claimed for this variation, and it is interpreted only as a possible trend toward improved surface oxygen exchange. The chemical surface-exchange coefficient K_chem_ reflects the kinetics of oxygen exchange between the gas phase and the solid surface. By contrast, the chemical diffusion coefficient (D_chem_), which mainly reflects chemical diffusion within the bulk, decreases from 5.9 × 10^−5^ cm^2^ s^−1^ for STFW to 1.31 × 10^−5^ cm^2^ s^−1^ for 30PCO-STFW. The substantially lower D_chem_ dominates the overall relaxation behavior and accounts for the slower approach to equilibrium of 30PCO-STFW. These results suggest that PCO modification primarily affects the surface and interfacial reaction environment rather than improving bulk oxygen transport [30,33,34,35].

The influence of PCO infiltration on the surface oxygen environment was further investigated by O 1s XPS, as shown in Figure 3d. The O 1s spectra of both STFW and 30PCO-STFW can be deconvoluted into three main components, which are assigned to lattice oxygen (O_lat_), adsorbed oxygen species (O_ads_), and hydroxyl-related surface species (O_H_), respectively. For pristine STFW, the relative fractions of O_lat_, O_ads_, and O_H_ are 41.9%, 49.0%, and 9.0%, respectively. After PCO infiltration, the O_lat_ contribution increases markedly to 58.5%, whereas the O_ads_ fraction decreases to 31.4%, accompanied by a slight increase in the O_H_ contribution to 10.2%. The pronounced redistribution of the O 1s components indicates that PCO infiltration substantially modifies the near-surface oxygen coordination environment of the STFW electrode. In particular, the increased O_lat_ contribution is consistent with the introduction of the fluorite PCO phase and the resulting reconstruction of the local oxygen environment at the PCO/STFW interface. Meanwhile, the decrease in the relative O_ads_ fraction should not be directly interpreted as a reduction in oxygen-vacancy concentration or surface reaction activity, because the individual O 1s components represent the static distribution of surface oxygen species and cannot uniquely quantify oxygen vacancies [36]. Instead, the simultaneous variation in O_lat_, O_ads_, and O_H_ demonstrates a substantial reorganization of the surface oxygen species after PCO modification. When considered together with the ECR results, this surface reconstruction becomes more meaningful. Although 30PCO-STFW exhibits a lower relative fraction of O_ads_, the fitted K_chem_ value does not decrease and instead shows a modest increase. This observation suggests that the static relative fraction of adsorbed oxygen species is not directly proportional to the dynamic surface-exchange response. Therefore, the electrochemical enhancement of 30PCO-STFW is more reasonably associated with regulation of the near-surface oxygen environment and surface/interfacial reaction chemistry rather than simply with an increased amount of adsorbed oxygen species.

Taken together, PCO infiltration decreases the bulk electrical conductivity and chemical diffusion coefficient, produces a modest change in the fitted surface-exchange coefficient, and induces a pronounced redistribution of the near-surface oxygen species.

### 3.2. Electrochemical Performance

#### 3.2.1. Symmetrical-Cell Performance

**Figure 4:**
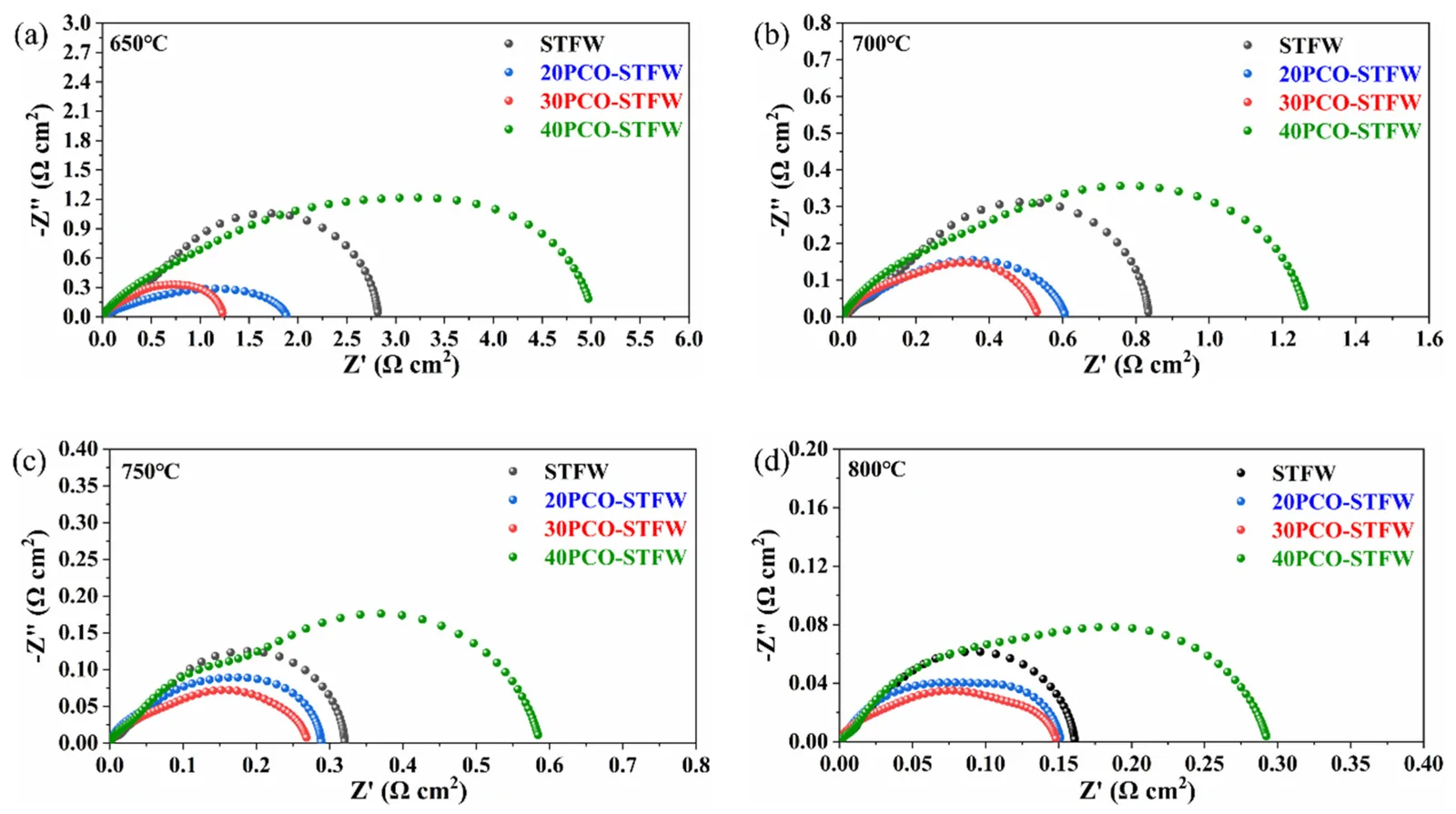
(**a**–**d**) EIS of STFW electrodes loaded with different amounts of PCO under 70% CO_2_/30% CO atmosphere at 650–800 °C.

To further evaluate the effect of PCO infiltration on the CO_2_ electrolysis reaction kinetics of the STFW fuel electrode, electrochemical impedance spectroscopy was performed on symmetrical cells with different PCO loading amounts under a 70% CO_2_/30% CO atmosphere. As shown in Figure 4a–d, the polarization resistance (Rp) of all samples decreases markedly with increasing temperature, indicating the thermally activated nature of the electrode reaction process. Compared with the pristine STFW electrode, an appropriate amount of PCO infiltration significantly reduces the electrode polarization resistance, suggesting that PCO introduction effectively improves the surface reaction kinetics of the STFW fuel electrode. Among all samples, 30PCO-STFW exhibits the lowest Rp over the entire temperature range. The Rp values of 30PCO-STFW are 1.23, 0.53, 0.26, and 0.14 Ω cm^2^ at 650, 700, 750, and 800 °C, respectively, corresponding to reductions of 56.23%, 36.14%, 18.75%, and 12.50% compared with pristine STFW. These results indicate that the PCO/STFW fluorite–perovskite heterointerface effectively promotes the surface reaction and interfacial transport processes involved in CO_2_ electrolysis. The larger relative reduction in Rp at 650 and 700 °C indicates that PCO modification is particularly beneficial under kinetically more demanding low-temperature conditions, where surface and interfacial processes generally become slower. In this temperature region, the surface and interfacial modification introduced by PCO may more effectively alleviate these kinetic limitations.

It is worth noting that a higher PCO loading does not necessarily lead to better electrochemical performance. At a relatively low infiltration amount, 20PCO-STFW shows a moderate decrease in Rp compared with STFW, but the relatively low PCO loading may provide insufficient surface and interfacial modification, resulting in only partial performance improvement. When the infiltration volume increases to 30 μL, an appropriate degree of PCO surface modification is achieved while the porous electrode architecture remains largely preserved, resulting in the lowest polarization resistance among the investigated samples. However, when the infiltration volume is further increased to 40 μL, the polarization resistance increases markedly and even exceeds that of pristine STFW. This deterioration may be associated with excessive PCO surface loading, which can partially obstruct gas-transport pathways and adversely affect electronic transport continuity within the composite electrode [17,20,21,22,23,24]. This interpretation is consistent with the general conductivity penalty associated with PCO incorporation observed for the optimized composite, highlighting the importance of optimizing the PCO loading to balance enhanced surface activity with maintained transport continuity.

**Figure 5:**
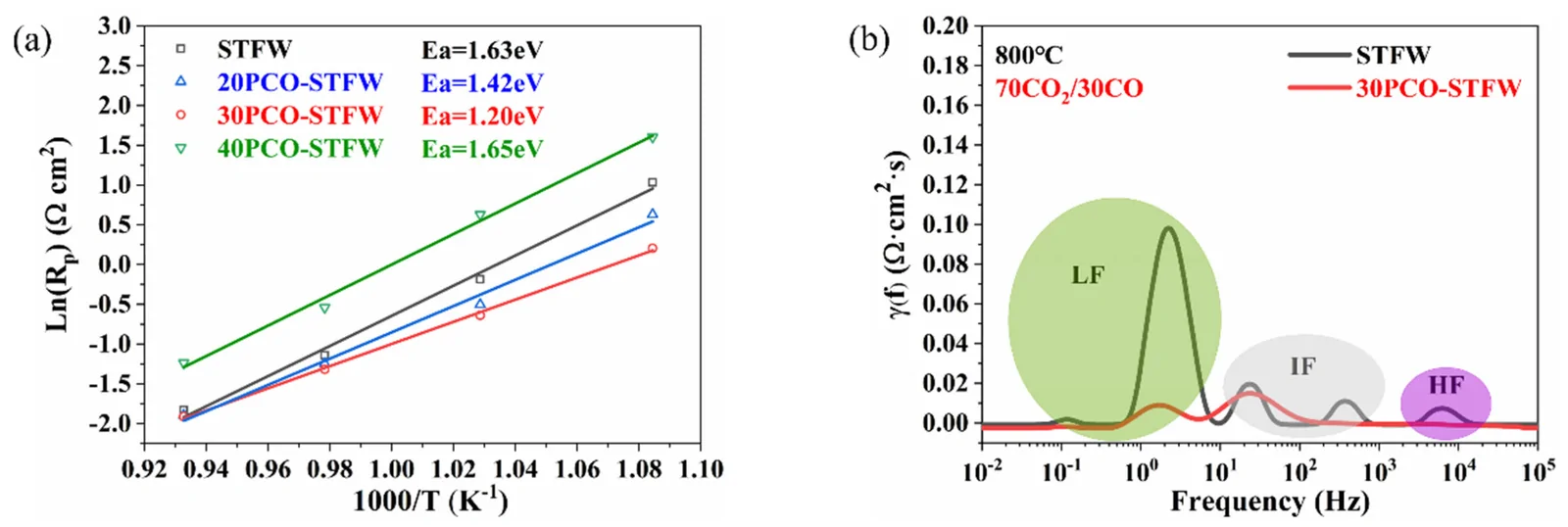
(**a**) Arrhenius plots of ln(Rp) versus 1000/T for STFW and PCO−STFW electrodes with different PCO loadings; (**b**) DRT profiles of STFW and 30PCO-STFW measured at 800 °C in 70% CO_2_/30% CO.

To further clarify the influence of PCO infiltration on the reaction energy barrier, Arrhenius fitting was performed using the Rp values obtained at different temperatures, as shown in Figure 5a. The apparent activation energy of the pristine STFW electrode is 1.63 eV, whereas those of 20PCO-STFW and 30PCO-STFW decrease to 1.42 and 1.20 eV, respectively. In particular, 30PCO-STFW exhibits the lowest activation energy, indicating that an appropriate amount of PCO can effectively reduce the kinetic barrier for CO_2_ electrolysis. By contrast, the activation energy of 40PCO-STFW increases to 1.65 eV, in agreement with its higher polarization resistance, further confirming that excessive PCO loading is detrimental to the electrode reaction process. Therefore, the superior performance of 30PCO-STFW does not simply arise from the addition of a secondary surface phase but is primarily associated with the synergistic promotion of CO_2_ adsorption/activation, interfacial charge transfer, and oxygen-ion migration at the PCO/STFW heterointerface.

To distinguish the contributions of different electrode reaction steps to the overall polarization resistance, distribution of relaxation times (DRT) analysis was performed on the impedance spectra of STFW and 30PCO-STFW at 800 °C. As shown in Figure 5b, the DRT profiles can be divided into three main response regions: high frequency (HF), intermediate frequency (IF), and low frequency (LF). In general, the HF region is mainly associated with interfacial charge transfer, the IF region is related to surface exchange, adsorbed-species conversion, and oxygen-ion migration, while the LF region is primarily attributed to slower surface and transport processes such as gas diffusion and CO_2_ adsorption/dissociation [37,38]. Compared with STFW, 30PCO-STFW shows suppressed DRT peak intensities across all three frequency regions, with the most significant decrease observed in the LF region. This indicates that PCO infiltration effectively alleviates the low-frequency limitation associated with CO_2_ adsorption/dissociation and gas-transport-related processes, while also reducing the kinetic contributions from interfacial charge-transfer and surface-reaction processes.

Combined with the preceding structural, physicochemical, and electrochemical analyses, these results show that PCO incorporation modifies the porous STFW electrode without destroying its overall porous architecture. The coexistence of the fluorite-type PCO phase and perovskite STFW phase provides additional interfacial contact within the composite electrode. Although the present SEM observations do not directly resolve the PCO/STFW interface at the nanoscale, the combined XRD, XPS, ECR, EIS, and DRT results support the interpretation that surface and interfacial regulation contributes to the reduced polarization resistance and apparent activation energy. Taken together, the EIS, Arrhenius, and DRT results indicate that an appropriate PCO loading effectively reduces the kinetic losses of the STFW electrode, with 30PCO-STFW exhibiting the most favorable overall electrode kinetics.

#### 3.2.2. Single-Cell Performance

**Figure 6:**
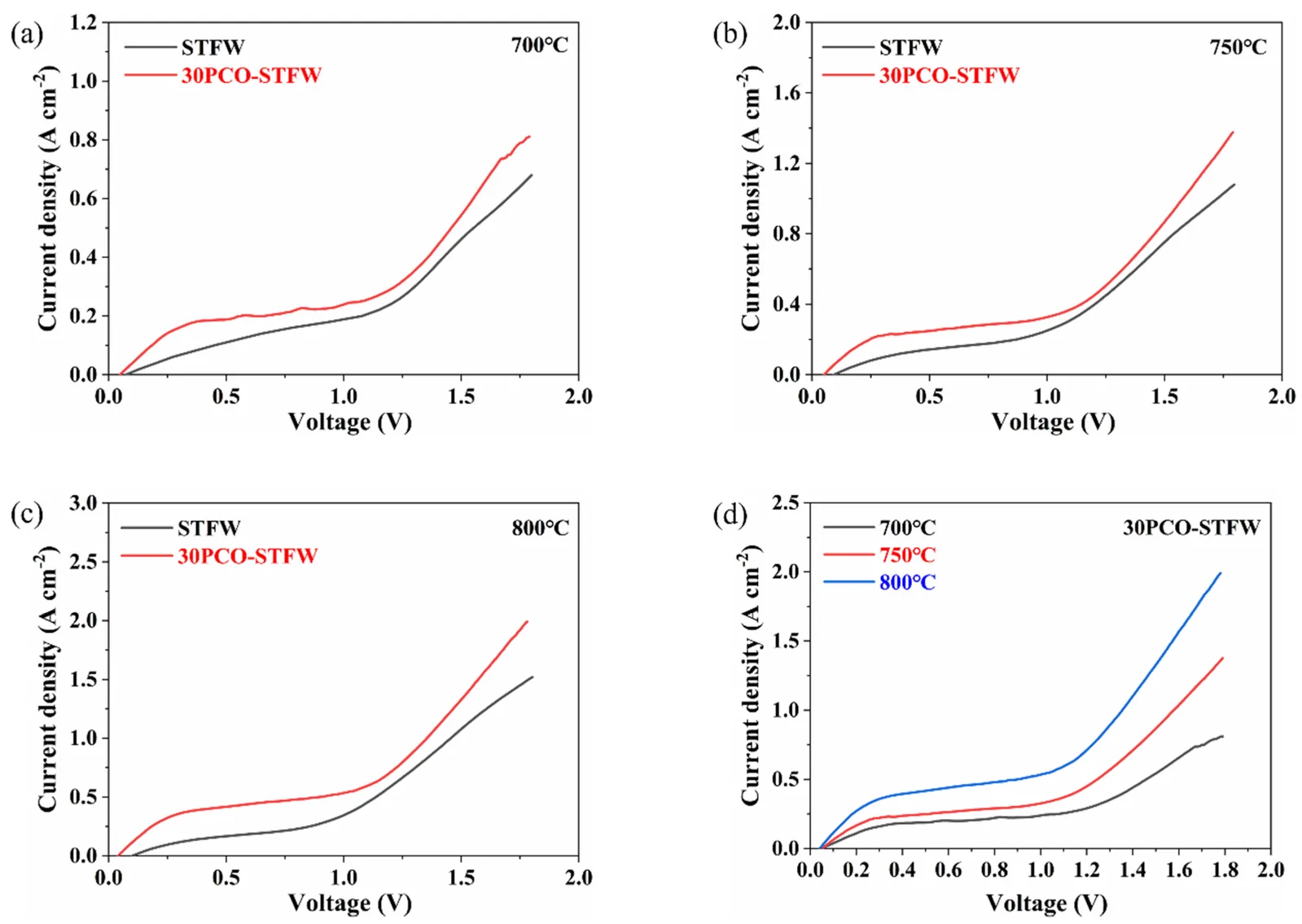
I−V curves of single cells with 30PCO-STFW and STFW fuel electrodes at (**a**) 700 °C, (**b**) 750 °C, and (**c**) 800 °C; (**d**) I−V curves of the single cell with the 30PCO−STFW fuel electrode at different temperatures.

In the symmetrical-cell measurements, 30PCO-STFW exhibits the lowest polarization resistance and apparent activation energy, indicating its superior fuel electrode reaction kinetics. To further verify its performance advantage under practical electrolysis conditions, single cells were fabricated using LSGM as the electrolyte, LSCF-GDC as the air electrode, and either STFW or 30PCO-STFW as the fuel electrode. CO_2_ electrolysis performance was then evaluated at 700–800 °C. Figure 6a–c shows the I–V curves of STFW and 30PCO-STFW single cells at different temperatures. The current density increases with applied voltage for both cells, while 30PCO-STFW consistently delivers a higher current response than pristine STFW. Compared with the pristine STFW cell, the 30PCO-STFW cell delivers higher current densities over the entire tested temperature range, demonstrating that the fluorite–perovskite heterointerfaces constructed by PCO infiltration effectively enhance the CO_2_ electrolysis activity of the fuel electrode. Specifically, at an applied voltage of 1.5 V, the current densities of the 30PCO-STFW cell reach 0.54, 0.87, and 1.33 A cm^−2^ at 700, 750, and 800 °C, respectively, whereas the corresponding values for the STFW cell are only 0.46, 0.74, and 1.08 A cm^−2^. These values represent performance enhancements of approximately 17%, 18%, and 23%, respectively.

Figure 6d further compares the I-V behavior of the 30PCO-STFW cell at different operating temperatures. As the temperature increases from 700 to 800 °C, the electrolysis current density increases significantly, indicating that elevated temperature facilitates CO_2_ adsorption/activation, charge transfer, and oxygen-ion migration. In particular, the rapid increase in current density at higher applied voltages suggests that the 30PCO-STFW composite fuel electrode can maintain fast CO_2_ reduction kinetics under a strong electrochemical driving force.

Combined with the preceding EIS, Arrhenius, DRT, ECR, and XPS analyses, the enhanced single-cell performance of 30PCO-STFW cannot be attributed to improved bulk transport, because both the electrical conductivity and chemical diffusion coefficient decrease after PCO incorporation. Instead, the electrochemical results suggest that PCO modification regulates the near-surface oxygen environment and the surface/interfacial reaction processes of the STFW electrode. The coexistence of the fluorite-type PCO phase and perovskite STFW phase introduces additional interfacial contact within the composite electrode, which may contribute to the improved reaction kinetics. Therefore, the performance enhancement is mainly associated with surface and interfacial kinetic regulation rather than bulk transport enhancement.

**Figure 7:**
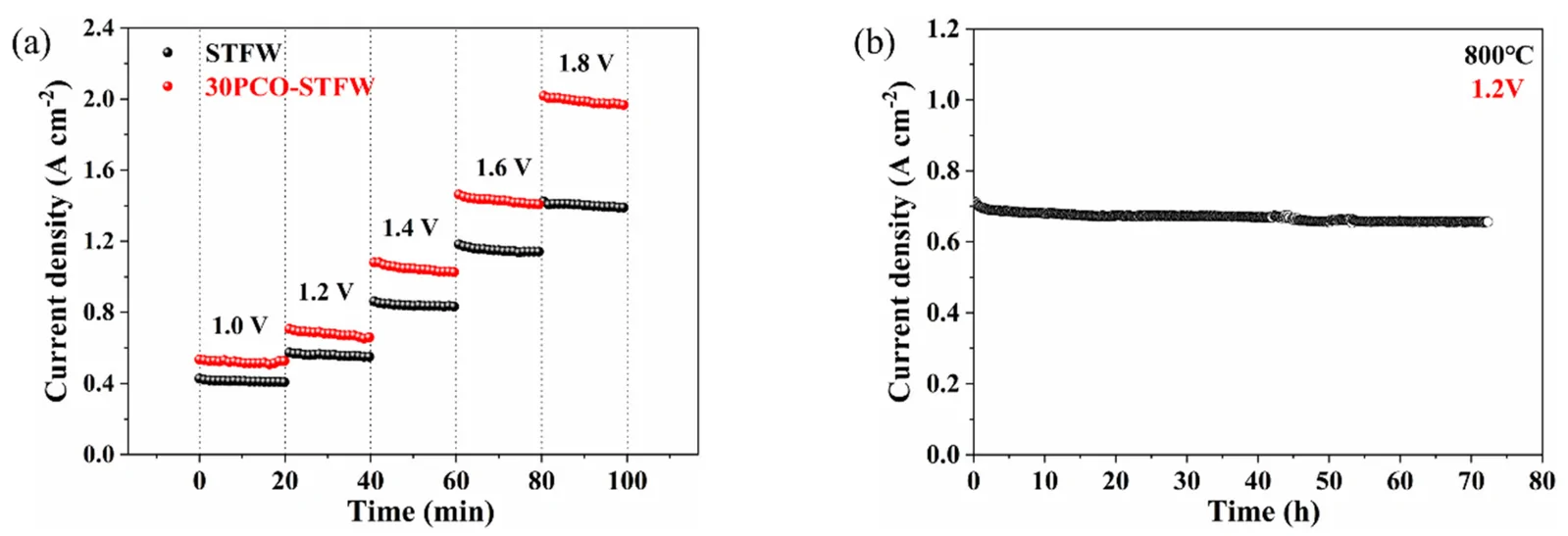
(**a**) Potentiostatic tests of the PCO−STFW and STFW single cells at 800 °C under applied voltages from 1.0 to 1.8 V. (**b**) Single−cell stability.

To further evaluate the electrochemical response and operational stability of the PCO-STFW fuel electrode under practical CO_2_ electrolysis conditions, potentiostatic measurements were performed on STFW and 30PCO-STFW single cells. As shown in Figure 7a, when the applied voltage is stepwise increased from 1.0 to 1.8 V, the current densities of both cells increase accordingly, indicating that the CO_2_ electrolysis reaction responds rapidly to the enhanced electrochemical driving force. Compared with the STFW cell, the 30PCO-STFW cell delivers higher current densities at each voltage step and maintains relatively stable output at each voltage plateau. This result further demonstrates that the PCO/STFW heterointerfaces constructed by PCO infiltration not only reduce the steady-state polarization resistance but also sustain fast CO_2_ reduction kinetics under dynamic voltage operation.

Short-term operational stability was further evaluated to assess the electrochemical stability of the 30PCO-STFW fuel electrode under CO_2_ electrolysis conditions. As shown in Figure 7b, the 30PCO-STFW single cell maintains a relatively stable current output during continuous operation at 800 °C and 1.2 V for 70 h. A slight decrease in current density is observed during the initial stage, followed by gradual stabilization, with the current density decreasing from approximately 0.70 to 0.65 A cm^−2^. This corresponds to a current retention of approximately 93% after 70 h and an average relative current decay rate of approximately 0.10% h^−1^, indicating acceptable short-term operational stability under CO_2_ electrolysis conditions. Nevertheless, the present 70 h test is insufficient to establish long-term durability, and longer-duration measurements are required for a comprehensive evaluation of long-term stability [7,9,16,17,18,19,20,21,22,23,24].

**Figure 8:**
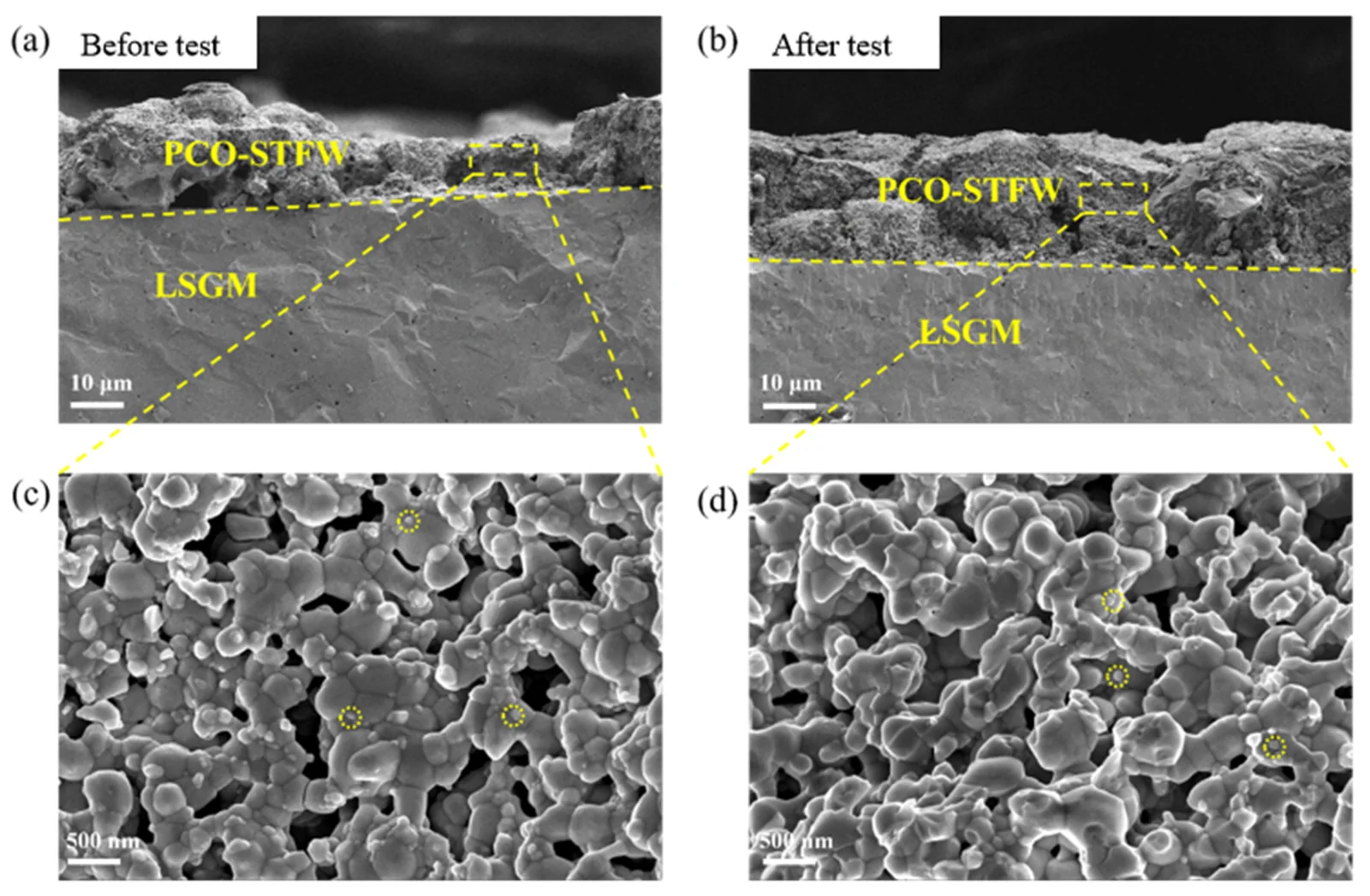
Microstructural characterization of the single cell before and after the stability test: (**a**) before testing; (**b**) after testing; magnified images of the electrode microstructure; (**c**) before testing; (**d**) after testing.

To further examine structural changes before and after the 70 h stability test, SEM was conducted on the cross-section and local fuel electrode morphology of the 30PCO-STFW single cell. As shown in Figure 8a,b, after 70 h of CO_2_ electrolysis, the PCO-STFW fuel electrode remains tightly bonded to the LSGM electrolyte, with no obvious interfacial cracking, delamination, or enlarged voids observed. The dense LSGM electrolyte layer is also well preserved after testing, indicating that the overall cell architecture remains structurally intact under the applied electrolysis conditions. In Figure 8c,d, a comparison of the local fuel electrode morphology before and after testing further reveals that the PCO-STFW electrode retains a continuously interconnected porous framework with open gas-transport channels, which is beneficial for CO_2_/CO diffusion and product removal. Meanwhile, slight particle growth and surface coarsening can be observed in some local regions, consistent with the mild current-density degradation during the 70 h CO_2_ electrolysis test. Nevertheless, the examined electrode regions remain porous and well connected, without severe densification, pore blockage, or interfacial delamination after the 70 h test.

**Figure 9:**
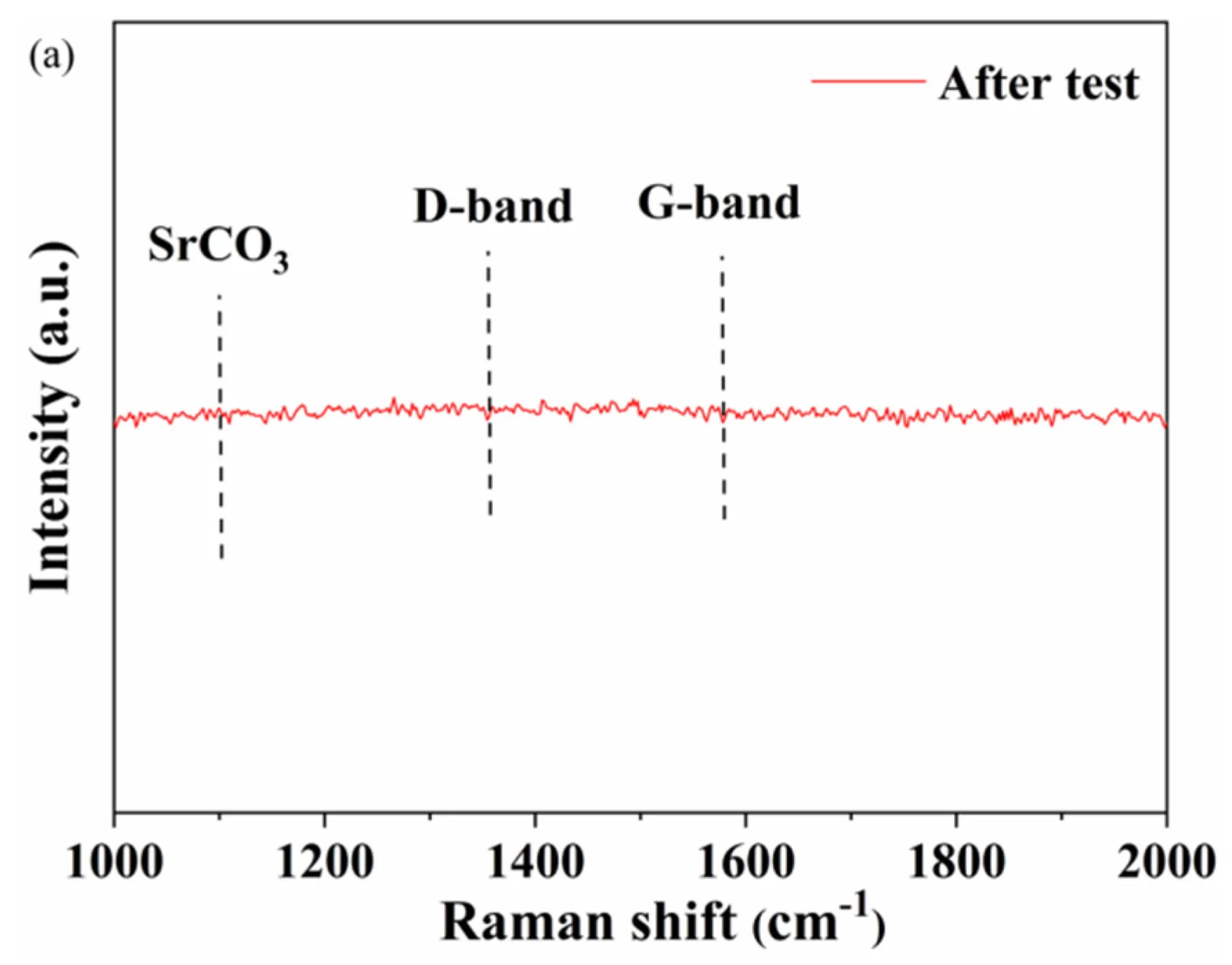
Raman spectrum of the PCO−STFW fuel electrode after the 70 h stability test.

Raman spectroscopy was further performed on the 30PCO-STFW electrode after the 70 h stability test to examine whether carbon deposition or carbonate accumulation occurred during CO_2_ electrolysis. As shown in Figure 9, no obvious SrCO_3_ characteristic peak at approximately 1100 cm^−1^ is observed after testing. In addition, the typical D band and G band of carbonaceous species at around 1338 and 1568 cm^−1^, respectively, are not detected [6,9,39]. These results indicate that no evident carbonate by-product accumulation or carbon deposition occurs on the 30PCO-STFW fuel electrode during CO_2_ electrolysis. Combined with the short-term operational stability test and post-test SEM observations, the PCO-STFW electrode not only maintains relatively stable current output and an intact electrode/electrolyte interface but also exhibits good resistance to carbon deposition.

Overall, the 30PCO-STFW fuel electrode achieves enhanced CO_2_ electrolysis activity while maintaining acceptable short-term structural and electrochemical stability. The electrode retains a porous architecture after the 70 h electrolysis test, while Raman spectroscopy shows no detectable carbon-related D or G bands. These results indicate that no severe structural degradation or carbon deposition occurs during the present short-term electrolysis test, although longer-duration measurements are required to evaluate long-term durability.

## 4. Conclusions

In this work, PCO was introduced onto the surface of the STFW fuel electrode through a surface infiltration strategy, constructing a stable PCO/STFW fluorite–perovskite composite fuel electrode. Structural characterization confirms that PCO infiltration does not disrupt the STFW perovskite backbone, while PCO remains as an independent fluorite phase and shows good chemical compatibility with the LSGM electrolyte. SEM observations show that the overall porous electrode architecture is largely preserved after PCO modification, although the PCO and STFW phases cannot be unambiguously distinguished from morphological contrast alone. Despite the decreased bulk electrical conductivity and chemical diffusion coefficient, PCO infiltration enhances surface oxygen exchange and modifies the near-surface oxygen environment, indicating that the performance improvement predominantly arises from surface and interfacial kinetic regulation rather than bulk transport enhancement. Electrochemical measurements demonstrate that an appropriate amount of PCO infiltration significantly improves the CO_2_ electrolysis reaction kinetics of the STFW fuel electrode. Among the investigated samples, 30PCO-STFW delivers the best performance, with a reduced polarization resistance of 0.14 Ω cm^2^ at 800 °C in 70% CO_2_/30% CO and a lowered apparent activation energy of 1.20 eV. DRT analysis reveals reduced kinetic contributions across the major frequency regions, with the most pronounced suppression occurring in the low-frequency response associated with CO_2_-related surface and gas-transport processes. A single cell employing 30PCO-STFW as the fuel electrode achieves an electrolysis current density of 1.33 A cm^−2^ at 800 °C and 1.5 V, and exhibits acceptable short-term operational stability during a 70 h test at 800 °C and 1.2 V, with approximately 93% current retention. Post-test SEM confirms the retained electrode/electrolyte architecture, while Raman spectroscopy reveals no detectable carbon-related D or G bands after the 70 h test. These findings highlight fluorite–perovskite heterointerface engineering via PCO infiltration as an effective route to overcome the sluggish surface reaction kinetics of SrTiO_3_-based SOEC fuel electrodes for efficient CO_2_ electrolysis.

## Data Availability

The original contributions presented in this study are included in the article. Further inquiries can be directed to the corresponding author.
